# Efficient Depth Enhancement Using a Combination of Color and Depth Information

**DOI:** 10.3390/s17071544

**Published:** 2017-07-01

**Authors:** Kyungjae Lee, Yuseok Ban, Sangyoun Lee

**Affiliations:** Department of Electrical and Electronic Engineering, Yonsei University, Seoul 03722, Korea; kjaelee@yonsei.ac.kr (K.L.); van@yonsei.ac.kr (Y.B.)

**Keywords:** depth enhancement, depth recovery, hole filling, image segmentation, RGB-D sensor

## Abstract

Studies on depth images containing three-dimensional information have been performed for many practical applications. However, the depth images acquired from depth sensors have inherent problems, such as missing values and noisy boundaries. These problems significantly affect the performance of applications that use a depth image as their input. This paper describes a depth enhancement algorithm based on a combination of color and depth information. To fill depth holes and recover object shapes, asynchronous cellular automata with neighborhood distance maps are used. Image segmentation and a weighted linear combination of spatial filtering algorithms are applied to extract object regions and fill disocclusion in the object regions. Experimental results on both real-world and public datasets show that the proposed method enhances the quality of the depth image with low computational complexity, outperforming conventional methods on a number of metrics. Furthermore, to verify the performance of the proposed method, we present stereoscopic images generated by the enhanced depth image to illustrate the improvement in quality.

## 1. Introduction

RGB-D sensors are used to identify color and depth simultaneously in real time. With the development of low-cost commercial RGB-D sensors such as Kinect and PrimeSense, computer vision technologies utilizing depth images or color and depth images have been used to develop many vision applications such as object tracking [1,2], pose estimation [3,4,5] for human-computer interaction (HCI), 3D modeling [6,7,8] and video surveillance [9,10,11].

The practical use of depth information is recognized as a key technology for many three-dimensional multimedia applications. Over the years, researchers have attempted to develop technologies that generate a high-quality three-dimensional view. Using depth information, high-quality three-dimensional images can be generated in the form of a stereoscopic image, which provides the necessary sense of reality [12]. Accordingly, extensive multimedia research based on depth information has been conducted, such as depth image-based rendering (DIBR) [12,13], free-viewpoint television (FTV) [14,15], augmented reality (AR) [16], virtual reality (VR) [17] and mixed reality (MR) [18].

**Figure 1:**
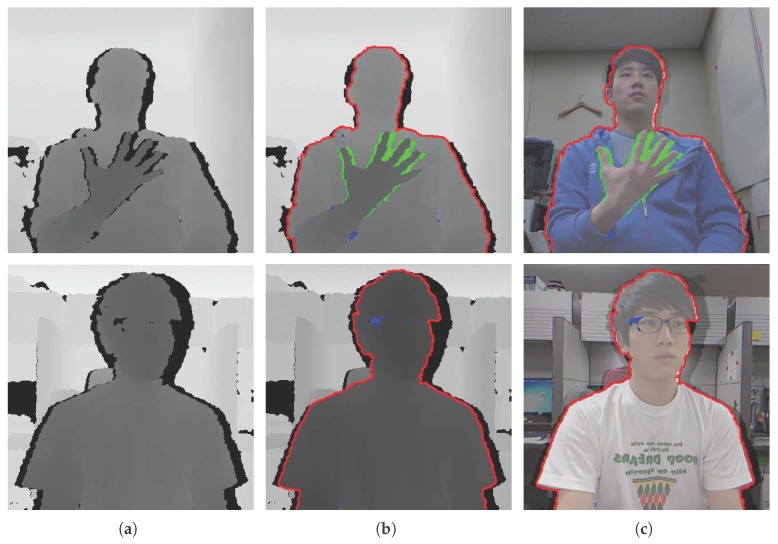
(**a**) The initial depth image; (**b**) the depth image with a colored marker; (**c**) the depth image overlaid with the color image with a colored marker. (The depth images are normalized and aligned with the color images. Blue, green and red markers indicate the first, second and third cases introduced in Section 2, respectively, and the black regions represent missing depth values.)

However, depth sensors that rely on infrared laser light with a speckle pattern (e.g., the Kinect sensor) suffer from missing or inaccurate depth information. These problems are caused by the incorrect matching of infrared patterns and a positional difference between the internal infrared sensors. Incorrect pattern matching yields numerous errors, such as optical noise, loss of depth values and flickering. Moreover, the different positions of the depth sensor, which is composed of an infrared projector and camera [19], mean that the rear regions may be occluded by the front object, making it difficult for depth information to be measured. In particular, there can be much noise around the object shape, as shown in Figure 1. The result is low-quality depth information, which makes it difficult to utilize the computer vision technologies [20,21,22]. For this reason, enhanced depth information is urgently required for applications.

A number of methods for enhancing the quality of depth information and overcoming the limitations of depth sensors have been proposed. Matyunin et al. [23] suggested an algorithm that uses color and motion information derived from the image sequences to fill occlusion regions of the depth image and improve the temporal stability. This algorithm can make depth images more stable, rectify errors and smooth the image. The confidence metric for motion vectors, spatial proximity and occlusion is highly dependent on the depth image. Fu et al. [24] proposed a divisive normalized bilateral filtering method that is a modification of the method proposed in [25], filling up the depth holes in the spatial domain and reducing the noise in the temporal domain. However, this approach leads to a blurry depth image and has a high computational cost. Joint bilateral-based methods, such as joint bilateral filter [26], joint bilateral upsampling [27] and weighted mode filtering [28], aim to improve the quality of the depth image by utilizing an aligned color and depth image. In these methods, the color image is used as a guide while the edges are preserved. Unfortunately, these methods frequently yield blurring effects and artifacts around boundaries in regions with large holes. Chan et al. [29] presented a noise-aware filtering method that enhances the quality and resolution of the depth image using an adaptive multi-lateral upsampling filter. However, this approach must be implemented on a GPU for real-time performance, and the parameters in the heuristic model must be set manually. Le et al. [30] suggested a directional joint bilateral filtering scheme based on [26]. This method fills the holes and suppresses the noise in the depth image using an adaptive directional filter that is adjusted on the basis of the edge direction of a color image. Although the directional joint bilateral filter performs well if the depth hole regions are located near the object boundaries, it is only applicable to four cases described by the edge directions. Lin et al. [31] proposed a method based on inpainting [32] for removing artifacts and padding the occlusions in a depth image. This approach is designed to inpaint the removed regions in a color image by assigning a priority to pixel locations and filling the removed regions based on these priorities. Though this method can eliminate depth noise and temporal variations and smooth inaccurate depth values, the processed depth values are changed from their original values. The computation time remains a problem for real-time applications. Gong et al. [33] incorporated guidance information from an aligned color image for depth inpainting by extending the inpainting model and the propagation strategy of the fast marching method [34]. This method reconstructs unknown regions simply but efficiently from the surrounding areas without additional information. However, this approach cannot convey texture information in the holes. Despite all efforts, these methods are time consuming and deliver blurry results, especially when the depth hole area is large.

To extract the object regions, many image segmentation techniques based on color information have been developed [35,36,37,38,39]. However, these methods suffer from challenging issues concerning illumination variations, shadows, and complex textures. RGB-D sensors have been employed to solve the problems of color-based image segmentation methods, because depth information is less affected by these issues, even if an image has shadows or complex textures [10]. One of the first approaches based on the fusion of color and depth information was developed by Gordon et al. [40], who presented the background model using an approximation of a 4D Gaussian mixture. Using a unimodal approximation, each image pixel is classified as foreground when the background exists in fewer sequences. However, the background model does not provide the correct fit when the background is dynamic and has various values per pixel. Schiller and Koch [41] proposed an object segmentation method by combining the segmentation of depth measurements with segmentation in the color domain using adaptive background mixture of Gaussian (MoG) models. To determine the depth reliability, the authors concluded that the amplitude information provided by the ToF camera is more effective than the depth variance. Fernandez-Sanchez et al. [9] generalized the background subtraction algorithm by fusing color and depth information based on a Codebook-based model [42]. In this method, the depth information is considered as the fourth channel of the codebook, and provides the bias for the foreground based on color information. This approach was extended [10] by building a late fusion mask technique based on morphological reconstruction to reduce the noise of the disparity estimated by stereo vision. Camplani and Salgado [43] suggested an efficient combination of classifiers based on a weighted average. One of the classifiers is based on the color features and the other is based on the depth feature, and the support of each classifier in the ensemble is adaptively modified by considering the foreground detected in the previous sequences and the edges of the color and depth images. del Blanco et al. [11] developed a Bayesian network using a background subtraction method based on [43] to distinguish foreground and background regions from depth sequence images. This method takes advantage of a spatial estimation model and an algorithm for predicting the changes of foreground depth distribution. However, many of these approaches are designed for video surveillance and require image sequence pairs. Moreover, the segmentation results still contain much noise in the foreground and background.

In this paper, we propose a high-performance, low-complexity algorithm based on color and depth information by using asynchronous cellular automata with neighborhood distance maps. Our approach aims to fill the missing depth holes and recover inaccurate object shapes in depth images. The proposed cellular automata-based depth recovery covers whole regions of the inaccurate and noisy depth image. Moreover, a weighted linear combination of spatial filtering algorithms is utilized to fill the inner depth holes in the object. Considering that humans are more sensitive to objects in an image than to its background [44], we focus on depth holes in the object regions. In general, depth hole filling methods based on color information utilize the color values of pixels that have a valid depth value to fill the neighboring depth holes. These methods fill the depth holes by calculating color-metric distances between the color pixel corresponding to the depth hole and the color pixels having a valid depth value. However, if the depth values of the reference pixels are inaccurate because of inherent depth sensor issues (e.g., misaligned color and depth values around the hand, as depicted in Figure 1c, top row), there is a high risk of incorrect depth values filling in the hole regions. To minimize this risk, we design a weighted linear combination of spatial filtering algorithms by reflecting the characteristics of the depth holes in the object (e.g., the blue and green markers in Figure 1). In this algorithm, depth information from the rear regions is used to fill the inner holes. To extract the object depth regions, we introduce an image segmentation algorithm using the connectivity values in the depth domain.

The remainder of this paper is organized as follows. Section 2 describes the proposed method in detail, including an introduction to image segmentation based on the depth domain, the procedure for filling inner depth holes in an object, and the recovery of a depth image. Section 3 presents our experimental results, and Section 4 states the conclusions from this research.

## 2. Proposed Methodology

In this section, we propose a method to enhance depth images using both color and depth information. The central premise is based on using a color image that has a relatively high resolution and more image information, such as texture and colors, than the depth image. The proposed calculations on the color image are intended to enhance the depth quality.

The problems with the images captured by depth sensors are as follows:

- Intermittent gaps in depth values in object regions, mainly because of reflections on the surface of the object (blue areas in Figure 1).
- Depth information of the rear regions cannot be estimated because the different positions of internal sensors in the depth sensor cause the front object to interfere with the depth measurement (green markers in Figure 1).
- Inaccuracies in the shape of objects compared to the actual scene. The depth value of an actual object consists of the object depth value (correct), background depth value (incorrect), and a missing depth value (incorrect) (red areas in Figure 1 show the inaccurate object boundaries).

**Figure 2:**
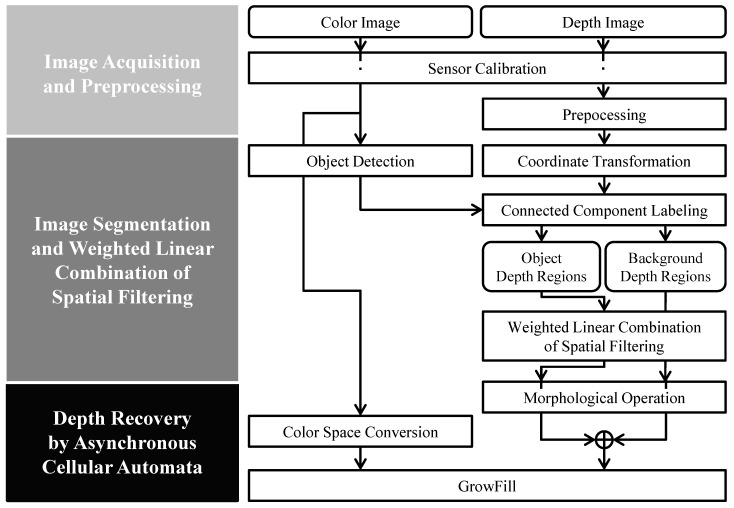
Flowchart of the proposed method.

In this study, we define an inner hole as the region with a missing depth value on account of gaps and interference from front objects, as stated above. Missing depth values are also called depth holes. To solve the problems of gaps and interference, inner holes are filled by a weighted linear combination of spatial filtering algorithms. In the case of shape inaccuracies, color and depth information is used to fill depth holes and recover the object shape. Our approach has three phases: image acquisition and preprocessing, image segmentation and weighted linear combination of spatial filtering, and depth recovery by asynchronous cellular automata (see Figure 2). In the first phase, the color and depth sensors are calibrated for the aligned color and depth image, and the depth image is filtered for the next phases. A morphological operation and spatial filtering are used to reduce and stabilize the depth noise. In the second phase, each object of the depth image is labeled according to the distribution, distance, and connectivity of depth values to separate the object regions and background. The inner holes in the object regions are filled using a weighted linear combination from the spatial filtering framework. The object and background depth regions are reduced using the morphological operation to recover accurate depth information in the next phase. The final phase uses a depth recovery algorithm to fill the remaining depth holes and refine the object boundary in the depth image. Details are explained in the following subsections.

### 2.1. Image Acquisition and Preprocessing

A color and depth image pair is acquired from the RGB-D sensor. As mentioned above, the image captured by the depth sensor contains noise, which may have an undesirable effect on the next phases. Hence, depth noise is reduced to stabilize the depth image.

**Figure 3:**
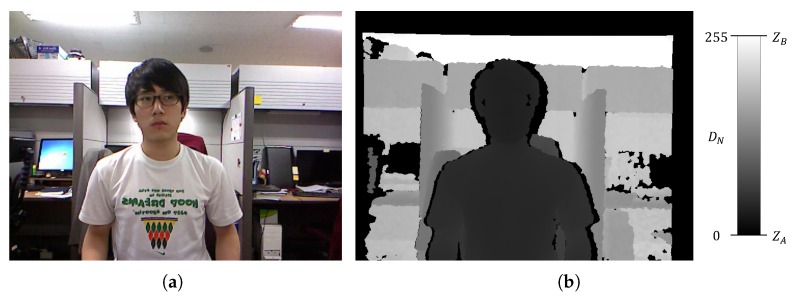
Aligned (**a**) color and (**b**) depth image pair. The depth images are normalized to $D_{N}$ (between 0 and 255).

To align the color and depth images, the color and depth sensors are calibrated using the camera geometrical model and calibration formulation [45]. Real depth values obtained from the depth sensor are normalized to the 8-bit range {0,255}, as shown in Figure 3b. The normalized depth values are utilized for object segmentation.

Equation (1) for the linear quantization of depth is implemented as the pixel value set to zero if the real depth value is less than $Z_{A}$, and the pixel value set to 255 if the real depth value higher than $Z_{B}$.

(1) $$D_{N}\left(i,j\right)=\left\{\begin{array}{cc} 0, & \mathrm{if}\;Z\left(i,j\right)<Z_{A}\\ 255\left(\frac{Z\left(i,j\right)-Z_{A}}{Z_{B}-Z_{A}}\right), & \mathrm{if}\;Z_{A}\leq Z\left(i,j\right)\leq Z_{B}\\ 255, & \mathrm{if}\;Z\left(i,j\right)>Z_{B} \end{array}\right.$$

where Z(i,j) and $D_{N}\left(i,j\right)$ are the real and eight-bit normalized depth values, respectively; i and j are the indices of the pixels in the depth image. $Z_{A}$ and $Z_{B}$ are the minimum (near) and maximum (far) real depth values, respectively. $Z_{A}$ and $Z_{B}$ are set within the reliable measurement range specified for the depth sensor. In this study, we set $Z_{A}=0.4$ m and $Z_{B}=3$ m in accordance with the Kinect specifications [46]. Thus, quantization darkens the near real depth values and brightens the far real depth values. Zero values represent missing depth values or real depth values of less than $Z_{A}$.

Morphological operations and a median filter are used to stabilize the initial depth image according to Equation (2). Before using the median filter, erosion is employed to reduce the size of the object regions. The median filter is then applied to smooth the image. Finally, a dilation process restores the object regions to their original size.

(2) $$D=\mathrm{median}\left(D_{N}⊖A\right)⊕B$$

where ⊖ and ⊕ denote erosion by pixel set A and dilation by pixel set B, respectively. D is the stabilized result of the normalized depth image ($D_{N}$). The preprocessing steps of erosion, median filtering, and dilation have the advantages of reducing the noise and smoothing the boundaries of objects in the depth image without changing their size. Furthermore, the size of depth regions can be reduced by changing the kernel size of the morphological operation when the object regions in the depth image exceed the boundary of the corresponding object in the color image.

### 2.2. Image Segmentation and Weighted Linear Combination of Spatial Filtering

First, the *x*-*y* pixel coordinates of the depth image are transformed into *x*-*D* coordinates by projecting all pixels in the pixel coordinate system onto the *x*-*D* coordinate system. Subsequently, a morphological operation is applied to connect neighboring valid points, and adjacent points on the transformed depth image are clustered by applying the connected component labeling algorithm [47]. The object regions in the depth domain are extracted by using an object detection method in the visual image. As a result, we can discriminate between the object and the background, and a weighted linear combination of spatial filtering algorithms is used to fill the inner depth holes in the object regions. A detailed explanation is provided in the following subsections.

**Figure 4:**
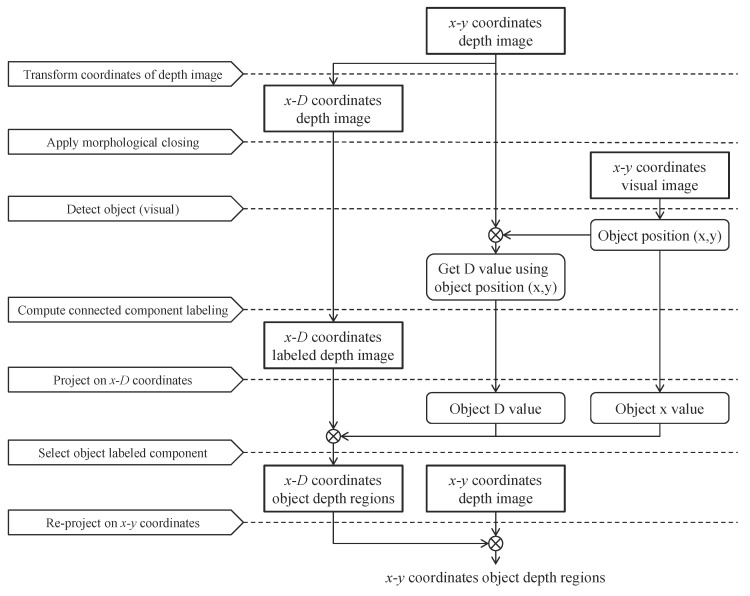
Flowchart for coordinate transformation and image segmentation process.

Figure 4 shows the flowchart of a coordinate transformation and image segmentation for a depth image. In this section, *x* and *y* denote the horizontal and vertical axes of the 2D pixel coordinates; *Z* and *D* indicate the real and normalized depth axes, respectively; and *X* is the horizontal axis of the 3D world coordinates.

#### 2.2.1. Coordinate Transformation of Depth Image

**Figure 5:**
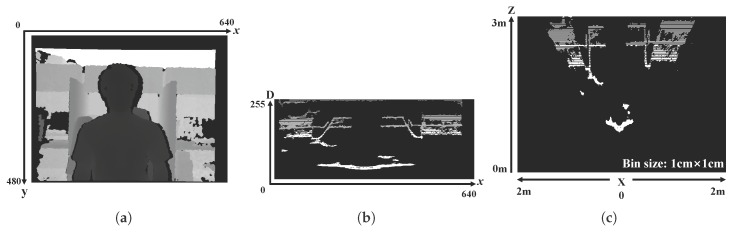
Depth image of (**a**) *x*-*y* coordinates; (**b**) *x*-*D* coordinates; and (**c**) *X*-*Z* coordinates; (**b**,**c**) are binarized; and (**c**) is normalized from mm to cm for visualization.

Each pixel of the color image (e.g., RGB color space) represents color information from the red, green, and blue channels, whereas each pixel of the depth image represents only depth information. This depth information can be transformed to another depth-based coordinate system. By using the D information instead of the information of *y* axis in *x*-*y* coordinates (Figure 5a), a new two-dimensional image can be represented with *x* and *D* domains as shown in Figure 5b, in which its pixel values represent accumulated D values on each column of *x* axis of the *x*-*y* coordinates. Accordingly, a depth image with *x*-*D* coordinates represents the three-dimensional information viewed from a top view. The *x*-*D* coordinate system of the depth image is useful for analysis because each object has similar depth values, which helps in the clustering of various objects and backgrounds.

The advantage of the *x*-*D* coordinate system (Figure 5b) over the *X*-*Z* coordinate system (Figure 5c) is that the *x*-*D* system produces salient objects from the normalized depth information. In addition, the sharing of the *x* axis allows us to project and re-project the images between *x*-*y* and *x*-*D* coordinates more easily than with *X*-*Z* coordinates.

#### 2.2.2. Image Segmentation in Depth Domain

To extract object regions that have connective pixels in terms of their normalized depth values and locations, a connected component labeling algorithm is applied to the depth image in *x*-*D* coordinates. Figure 5b shows that the pixels of each object are close together. The morphological operation of closing is performed to reinforce the connectivity of the objects.

**Figure 6:**
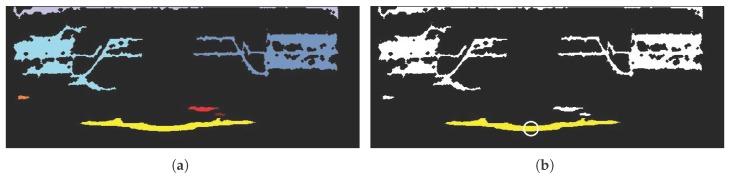
(**a**) Connected component labeling result (each colored marker involves discriminating objects); (**b**) result of object selection (circle indicates detected position).

**Figure 7:**
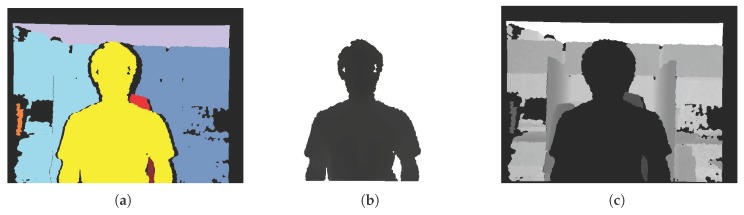
(**a**) Labeled depth image (colored markers correspond to connected component labeling results in Figure 6a); Extracted (**b**) object and (**c**) background regions of the depth image in *x*-*y* coordinates.

After closing the depth image in *x*-*D* coordinates, the connected components are labeled. Figure 6a shows an example of the connected component labeling. In this figure, the labeled objects are marked in different colors, wherein the values of the pixels are binarized. To extract one of the labeled objects as described in Figure 6a, object detection is applied to the color image. In this study, a pre-trained object detector [48] based on [49] is employed. From this object detection method, we obtain the depth value by using the detected position (x,y). This approach facilitates object selection that matches the detected location (indicated by the circle in Figure 6b) by being projected on the depth image in *x*-*D* coordinates. After object selection in *x*-*D* coordinates, we extract the object regions (Figure 7b) in the *x*-*y* coordinates by re-projecting the *x*-*D* coordinates information onto the depth image in *x*-*y* coordinates. Other regions are considered to be the background (Figure 7c).

#### 2.2.3. Weighted Linear Combination of Spatial Filtering for Inner Hole Filling

Depth sensors cannot measure depth information in regions of shadow and in the background. Regions of shadow are generally caused by objects in front, which is a geometrical limitation of depth sensors. These sensors consist of an infrared projector and an infrared camera at different positions. Accordingly, the different views of these compositions inevitably create problems such as inner holes on the boundary between the front and rear regions (the green areas in Figure 1b,c). Moreover, technical issues with depth sensors generate noise, i.e., reflection errors on a surface in which depth values cannot be measured (the blue areas in Figure 1b,c). To solve these problems, we propose a weighted linear combination of spatial filtering algorithms. The weighted linear combination is composed of the weighted sum of two terms, one related to the depth information of segmented depth regions and the other related to the depth information in the vicinity of inner holes, as shown in Equation (3).

(3) $$\begin{array}{c} H=\alpha \times \mathrm{mean}\left(Z_{seg}\right)+\beta \times Z_{N}\\ N=\underset{n\in k}{\mathrm{argmax}(Z_{n})} \end{array}$$

where H denotes inner hole pixels and Z is a real depth value. $Z_{\mathrm{seg}}$ denotes pixels in segmented depth regions. α and β are the weights of each term, with α+β=1. n indicates the searching mask size of surrounding pixels at the inner hole and k is the index of n. α, β and n are empirically determined according to the problem being considered.

From the mean real depth value of segmented depth regions and the maximum (far) real depth value of surrounding inner holes, the inner holes in the segmented regions are filled using the above equation. To compute real depth information, the equation uses real depth values. The mean depth value of the segmented depth regions is used to balance the depth biases of the holes, and the maximum real depth value surrounding the inner hole is used to account for depth similarities in the rear regions. Inner holes in the rear regions are mainly caused by the front objects. Hence, the depth values of the front regions are not considered. Therefore, the mean depth value of the segmented depth regions reflects global properties of the segmented depth regions, and the maximum real value reflects local properties of inner holes in the segmented regions.

### 2.3. Depth Recovery by Asynchronous Cellular Automata

To fill the depth holes and recover depth information for distorted object shapes in a depth image (the red areas in Figure 1b,c), we propose a depth recovery method inspired by [36] based on cellular automata [50]. Cellular automata are described by a triplet A=(S,N,δ) that reflects a discrete model in both space and time. For each cell, *S* indicates the state set and *N* is the neighborhood system, which is defined as the relationship between the specified cell and the surrounding cells (the von Neumann neighborhood (4-connected) or Moore neighborhood (8-connected) is generally used). δ indicates a local transition function that defines the rules for calculating the next state of each cell. The next state is determined from the current state of the cell and its neighboring cells.

In our proposal, asynchronous cellular automata (ACA) are applied. The ACA change states immediately, regardless of the processing steps, to reduce the number of iterations and computation time. In contrast, synchronous cellular automata (SCA) maintain their current states until the operation of the current step has been completed, and then change states simultaneously before the next step starts. The maximum strength value is given to pixels that have depth values. Conversely, pixels in depth holes are assigned the minimum strength value. These pixels are filled by taking advantage of the feature vectors given by the pixel values in a given color space, strength values of these pixels, and the transition function. The feature vectors of an input image do not change at all times. Therefore, it is unnecessary to repeatedly calculate the distance between the feature vectors of the current cell and its neighboring cells in every step. Finally, we change the RGB color space to the Lab color space to improve the performance of the algorithm. The pixel values represented in a given color space are considered as feature vectors. The details are explained in the following subsections.

#### 2.3.1. Asynchronous Cellular Automata

In an SCA system, all cells have the same state during the computation in each step. When a local transition function is applied to all cells in the current step, the states are updated simultaneously before the next step starts. Therefore, the states of time *t* and time t+1 are independent of each other. In other words, the result of the local transition at time *t* has no effect on other cells at the same time.

In the ACA system applied in the proposed method, however, the states change immediately when the local transition function is computed. The results of this local transition have an effect on the other cells, regardless of the step. Thus, an algorithm that spreads the state of the cell to the neighborhood can be efficiently represented by ACA. Using ACA in place of SCA reduces the number of iterations, and thus the computation time.

**Figure 8:**
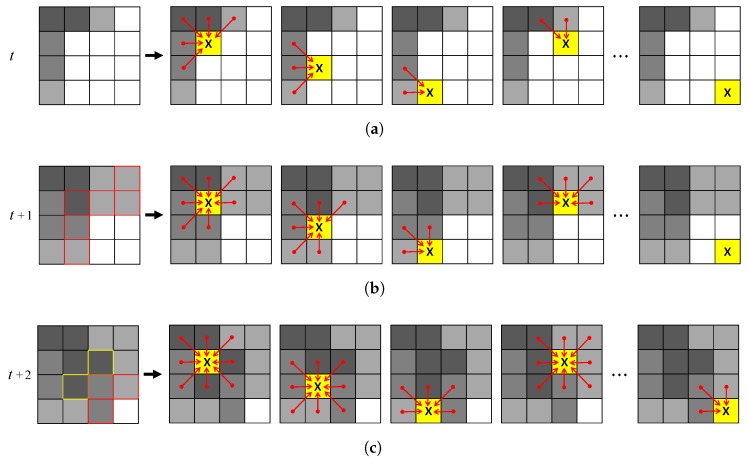
Cell evolution steps by SCA. (**a**) is at time *t*; (**b**) is at time t+1; and (**c**) is at time t+2. The first column shows the initial cell state at the time. (The area in yellow marked *X* indicates the current defender and the red arrow is the direction of attack on the defender by its neighboring cell, represented as the attacker. The rectangular areas in red and yellow indicate that the cell state has changed.)

**Figure 9:**
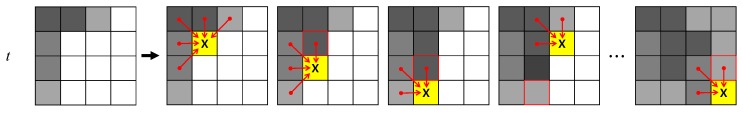
Cell evolution step by ACA. The first column is the initial cell state at time *t*. (The yellow marker denoted as *X* indicates the current defender and the red arrow is the direction of attack by its neighboring cell, represented as the attacker. The rectangular areas in red indicate that the cell state has changed.)

In this study, we adopted a vertical scan order as shown in Figure 8 and Figure 9. Figure 8 illustrates the cell evolution steps given by SCA. The current defender (colored yellow and marked *X* in Figure 8) does not change state until the current time step has been completed, although the defender has been conquered by the attacker and will be changed to the attacker’s state. The defenders’ states are updated simultaneously at the end of the current time. For instance, although the empty cells will be changed by the attackers, the empty state cells are not changed in the current time and have no effect on neighboring cells, as shown in Figure 8. In contrast, the current defender (colored yellow and marked *X* in Figure 9) changes state immediately when conquered by the attacker in the ACA system. The empty state cells immediately affect the neighboring cells when the state has changed, as shown in Figure 9, which illustrates the cell evolution under ACA. Comparing Figure 8 with Figure 9, the result that requires three steps for SCA takes only one step for ACA.

#### 2.3.2. Depth Recovery by Cellular Automata

To estimate a depth value and refine an object shape, we focus on the strength and feature vectors of cells. The cellular space P is defined by the image and each pixel is considered as a cell. For each cell *p* in P, the cell state $S_{p}$ has four terms $\left(d_{p},\vec{C_{p}},\theta_{p},b_{p}\right)$, where $d_{p}$ is a depth value, $\vec{C_{p}}$ is a feature vector, $\theta_{p}$ is a strength, and $b_{p}$ is a Boolean flag. The depth value $d_{p}$, strength $\theta_{p}$, and flag $b_{p}$ are defined by the depth image. The feature vector $C_{p}$ is defined by the color image. We assume that $\theta_{p}\in \left[0,1\right]$. If cell *p* has a valid depth value, then $\theta_{p}$ is set to the maximum value of 1 and $b_{p}$ is set to true. If cell *p* has an invalid depth value, $\theta_{p}$ and $b_{p}$ are set to zero and false, respectively. The Boolean flag $b_{p}$ indicates whether cell *p* has any depth value on the input depth image.

Algorithm 1 (Lines 6–28) depicts the entire process of the depth recovery method. To explain our method using a biological metaphor, a bacterium *p* (attacker) attacks its neighboring bacteria N(p) (defenders) using an attack force. The attack force is defined by the product of the strength $\theta_{p}$ of the attacker and the value obtained from Equation (4), expressed as follows [36].

(4) $$g\left(x\right)=1-\frac{x}{max{∥\vec{C}∥}_{2}}$$

in which *x* is the distance value between the feature vectors of attacker $\vec{C_{p}}$ and defender $\vec{C_{q}}$ as the output of Equation (7), and $\vec{C}$ is the feature vector. The function g(x) is a monotonously decreasing function with a minimum value of zero and a maximum value of one.

**Algorithm 1** Depth recovery by asynchronous cellular automata.**Input:** color image: $I_{c}∋\vec{C}$; depth image: $I_{d}∋d$;**Output:** enhanced depth image: $I_{d}∋d$;**Initialize:** condition flag: k←true;1:**for**
∀p∈P
**do**2:  **for**
∀q∈N(P)
**do**3:  ${\vec{NDM}}_{p,q}\leftarrow g\left({∥\vec{C_{p}}-\vec{C_{q}}∥}_{2}\right)$;4:  **end for**5:**end for**6:**for**
∀p∈P
**do**
7:  **if**
$d_{p}\neq 0$
**then**8:   $\theta_{p}\leftarrow 1$;9:   $b_{p}\leftarrow true$;10:  **else**11:   $\theta_{p}\leftarrow 0$;12:   $b_{p}\leftarrow false$;13:  **end if**14:**end for**15:**while**
k=true
**do**
16:  k←false;17:  **for**
∀p∈P
**do**18:   **if**
$b_{p}\neq true$
**then**19:    **for**
∀q∈N(p)
**do**
20:     **if**
${\vec{NDM}}_{p,q}\cdot \theta_{q}>\theta_{p}$
**then**21:      $d_{p}\leftarrow d_{q}$; 22:      $\theta_{p}\leftarrow {\vec{NDM}}_{p,q}\cdot \theta_{q}$;23:      k←true;24:     **end if**25:    **end for**26:   **end if**27:  **end for**28:**end while**

If the attack force is greater than the strength $\theta_{q}$ of the defender, the depth value $d_{q}$ and the strength $\theta_{q}$ of the defender are replaced by the attacker’s depth value $d_{p}$ and the attack force, respectively. When the replaced bacteria attack their neighboring defenders, they use the changed values immediately, regardless of the step. Only those bacteria that have a false flag ($b_{p}=false$) are repeatedly attacked. These operations are repeated until there is no change in the state of the cells. In this iterative process, the holes are filled by spreading the bacteria. For this reason, we called this method “GrowFill”. The computational complexity of GrowFill is O(snk), where *s* is the number of invalid pixels in the input depth image, *n* is the size of the neighborhood system, and *k* is the number of iterations.

#### 2.3.3. Neighborhood Distance Map

The steps involved in calculating the evolution of automata are continuously processed until the stable condition is reached. Equation (5) calculates the Euclidean distance between the feature vector of the current cell *p* and that of its neighboring cell *q*:

(5) $${∥\vec{C_{p}}-\vec{C_{q}}∥}_{2}=\sqrt{{\left(R_{p}-R_{q}\right)}^{2}+{\left(G_{p}-G_{q}\right)}^{2}+{\left(B_{p}-B_{q}\right)}^{2}}$$

where $\vec{C}$ is the feature vector of a specific pixel, which includes visual information. If the RGB color space is used for the feature vector, *R*, *G*, and *B* are the values of the red, green, and blue channels, respectively, as described in Equation (5). *p* is the pixel indicating the current cell and *q* is a pixel in the neighborhood of *p*.

The feature vector is indicated by pixel information from a color image. When the algorithm is executed, however, the feature vectors do not change until the end. The color image is a hard constraint, because the visual information does not change while the algorithm is being processed. Hence, the distance calculated between two feature vectors does not change, and there is no need to repeat the distance calculations at every step. Therefore, the neighborhood distance map can be generated before entering the automata evolution steps and used to find the necessary distances.

(6) $$\begin{array}{c} {\vec{NDM}}_{p,q}=g\left({∥\vec{C_{p}}-\vec{C_{q}}∥}_{2}\right)=1-\frac{{∥\vec{C_{p}}-\vec{C_{q}}∥}_{2}}{max{∥\vec{C}∥}_{2}} \end{array}$$

in which ${\vec{NDM}}_{p,q}$ is the neighborhood distance map (NDM). NDMs are generated before starting the evolution steps in Algorithm 1 (Lines 1–5). After the NDMs have been generated, they are used in every iterative step (Algorithm 1 (Lines 15–28)). As a result, during the operation of the algorithm, Equation (6) is not calculated in each iteration process.

#### 2.3.4. Lab Color Space

The RGB color space is commonly used to calculate the color-metric distance between feature vectors. Although the RGB color space is designed for hardware-oriented systems and is convenient for representing colors, it is not useful for object specification and recognition [51] and is not similar to the human perception of colors [52]. In contrast, the Lab color space is known to give a good representation of human color perception and is widely used for the evaluation of color differences and color matching systems [51]. Therefore, we use the Lab color space in the proposed algorithm.

Equation (7) is used to calculate the distance between feature vectors in our method.

(7) $${∥\vec{C_{p}}-\vec{C_{q}}∥}_{2}=\sqrt{{\left(L_{p}-L_{q}\right)}^{2}+{\left(a_{p}-a_{q}\right)}^{2}+{\left(b_{p}-b_{q}\right)}^{2}}$$

where $\vec{C}$ is a feature vector and L·a·b denotes the *L*, *a*, and *b* channel values. *p* is the pixel indicating the current cell, and *q* is a pixel in the neighborhood of *p*.

## 3. Experiments and Discussion

To validate our proposed method, we conducted a series of experiments on real-world Kinect datasets and the Tsukuba Stereo Dataset [53,54]. For the real-world datasets, we captured color and depth image pairs using the Kinect and obtained a public Kinect dataset [9,43,55]. The experimental results have been compared with state-of-the-art methods. All experiments were conducted on a desktop computer with Intel i7-3770 3.4 GHz and 16 GB RAM.

The experiments were as follows:

- Object segmentation (quantitative and qualitative evaluations).
- Inner hole filling (qualitative evaluation).
- Depth recovery (quantitative and qualitative evaluations).
- ACA, NDMs, and Lab color space on the proposed method (quantitative evaluation).
- Enhanced depth images and a practical application of the proposed method.

We evaluated the performance of the object segmentation method with Fernandez’s Kinect dataset [9] and compared our method with the mixture of Gaussians based on color and depth (MOG4D) [41], the codebook [42] based on depth (CB1D) and based on color and depth (CB4D), and the depth-extended codebook (DECB) [9].

To evaluate the results, the following measures are used:

- True positive (TP): the sum of foreground classified as foreground.
- True negative (TN): the sum of background classified as background.
- False positive (FP): the sum of background misclassified as foreground.
- False negative (FN): the sum of foreground misclassified as background.
- Precision (P): the proportion of TP and the total classified as foreground, $P=\frac{TP}{TP+FP}$.
- Recall (R): the proportion of TP and the ground truth, $R=\frac{TP}{TP+FN}$.
- $F_{1}$ score: the harmonic mean of precision and recall, $F_{1}=2\cdot \frac{P\cdot R}{P+R}$.

$F_{1}$ ranges from 0–1, with higher values indicating better performance.

**Figure 10:**
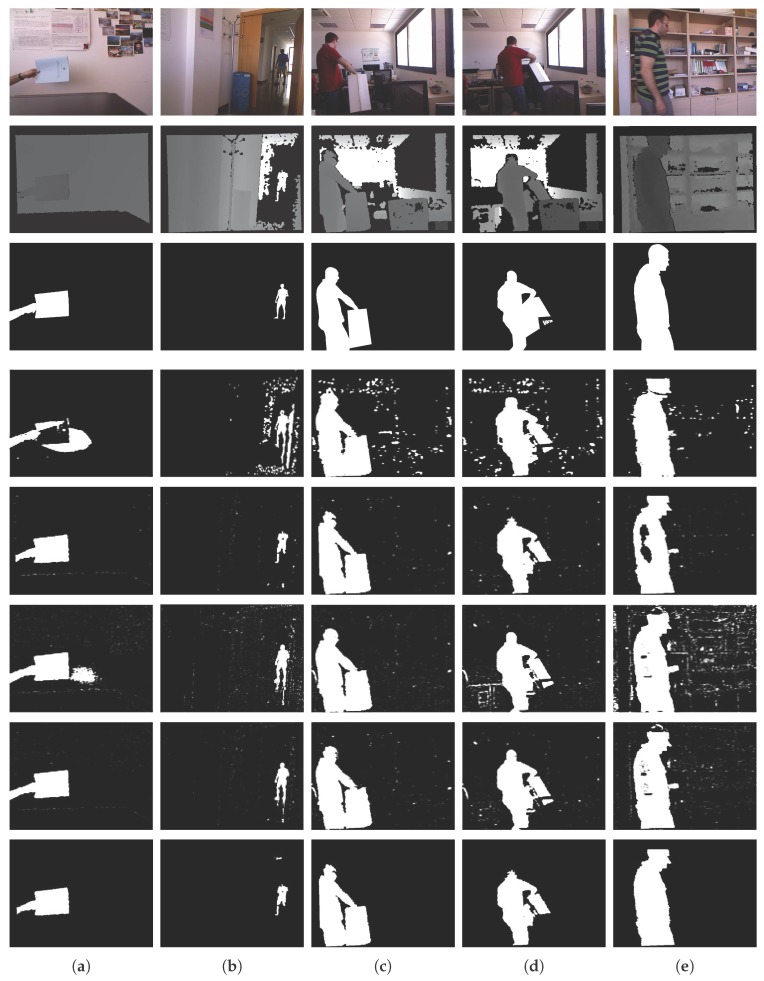
Experimental results using Fernandez’s Kinect dataset ((**a**–**e**) indicate Wall #93; Hallway #120; Chair Box #278, #286; Shelves #197, respectively). Rows 1–3 are the color images, depth images, and ground truth, respectively. Rows 4–8 present the results given by MOG4D, CB1D, CB4D, DECB, and the proposed method, respectively.

Fernandez’s Kinect dataset [9] provides image pairs including color, depth, and ground truth images for the foreground. As our proposed method focuses on single object, five different image pairs (Wall #93, Hallway #120, Chair Box #278 and #286, Shelves #197) were selected for the quantitative and qualitative tests. Following the literature, we compare the results reported in [9], as shown in Table 1 and Figure 10. A pre-trained body [56] and hand [57] detector were used as the object detector in our algorithm.

Table 1 presents the $F_{1}$ scores. Our method outperforms MOG4D, CB1D, and CB4D, and has very similar performance to DECB. From Figure 10, we can observe that all the compared methods generate much noise on the whole image. The DECB results, which give an average $F_{1}$ score that is 0.008 higher than that of our method, also contain much more noise than the image given by our algorithm. In particular, none of the compared methods can extract object regions that have the depth values of the depth image, as shown in Figure 10e. As the results are used for the following depth recovery algorithms, all the depth regions of the object should be extracted. Otherwise, the actual depth information may be distorted. In addition, when a region with no assigned depth is generated as a segmentation result, the region cannot be estimated in the following algorithms. The purpose of the segmentation at this stage is to extract only the object regions that have actual depth values to fill depth holes or manipulate the object boundary to recover depth values. Therefore, the object segmentation results should be object-oriented and the noise level should be low. Our method is best suited for this purpose.

**Figure 11:**
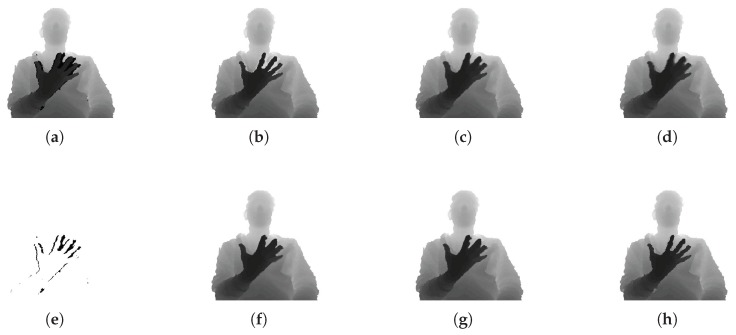
(**a**,**e**) are the segmented depth regions and masking region indicating inner depth holes, respectively; The others show the experimental results of the inner hole filling methods; (**b**) method based on [58]; (**c**) method in [59]; (**d**) method in [34]; (**f**) method in [26]; (**g**) method in [33]; (**h**) proposed method. (The contrast of the depth images has been adjusted for visualization.)

The following describes the performance of the inner hole filling methods, as shown in Figure 11. To evaluate the performance of inner hole filling, we collected color and depth image pairs acquired by the Kinect sensor in an indoor environment. As in Figure 11e, inner holes exist in the rear object (body) as a result of the front object (hand) in the segmented regions. The results of inner hole filling by the proposed method are compared to those of five previous methods: flood-fill based on morphological reconstruction [58], Navier–Stokes-based inpainting [59], fast marching inpainting [34], joint bilateral filtering [26], and guided depth inpainting followed by guided filtering [33]. We set n=23, α=0.3, and β=0.7 in Equation (3) for the proposed method, and set the radius value to 11, $\sigma_{d}=2$, and $\sigma_{c}=10$ for the methods in [26,33,34,59], as per the values recommended in [33].

From the results of the methods in [26,33,34,59], we can easily observe that the depth values in the inner holes are filled by the depth values of both front and rear objects idirectionally, so that the filled regions are blurred and have incorrect depth values. The methods in [26,33] use both the color and depth images. In these methods, the hole regions of the rear object are affected by the front depth values when the inner holes are filled based on color information. This is because the limitations of the depth sensor cause the depth and color regions of the object to be imprecisely matched. In the case of [34,59], which use only depth information, the blur effect is inevitable because the information on the boundary is initially unknown. In contrast, the method based on [58] and the proposed method fill the holes without spreading the depth values of the front object or blurring the output. The difference is that the method based on [58] fills the holes with the same depth value per hole, which results in a dissimilarity between the filled and actual depth values, whereas the proposed method fills the holes with similar depth values to the actual depth values. The proposed method considers the characteristics of the inner holes and fills them with similar depth values as the rear object without expanding the depth values of the front object. As a result, the proposed method gives the best results among all the methods compared in this experiment.

**Figure 12:**
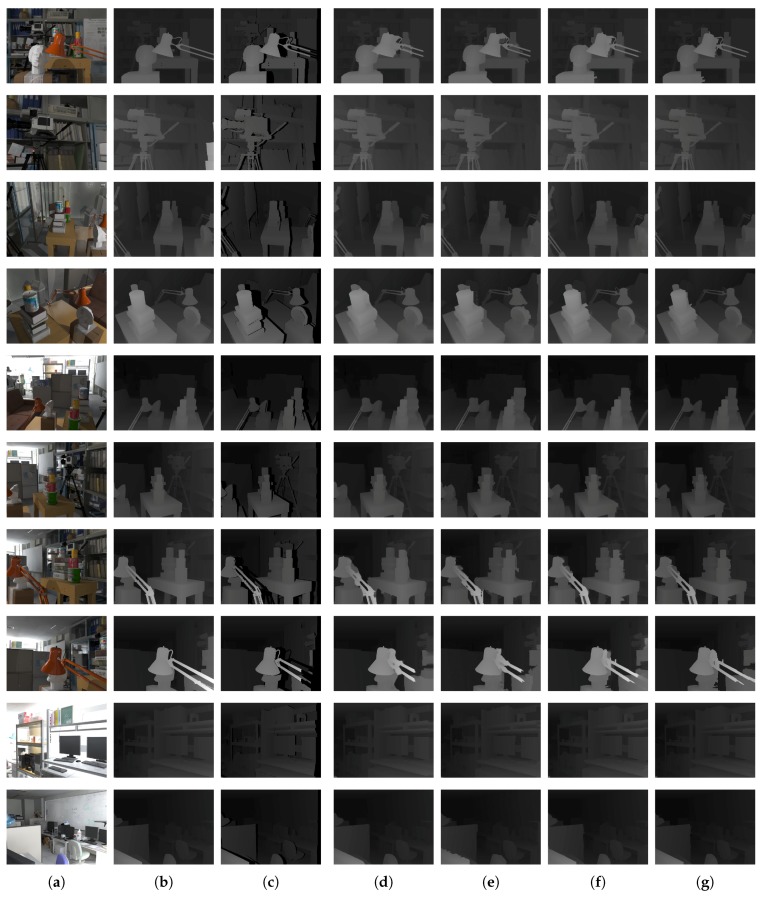
Experimental results using the Tsukuba Stereo Dataset (# 001; # 214; # 291; # 347; # 459; # 481; # 509; # 525; # 715; # 991). (**a**–**c**) are the color, ground truth of depth, and depth images, respectively; (**d**) is the method in [34]; (**e**) is the method in [31]; (**f**) is the method in [33]; and (**g**) is the proposed method.

**Figure 13:**
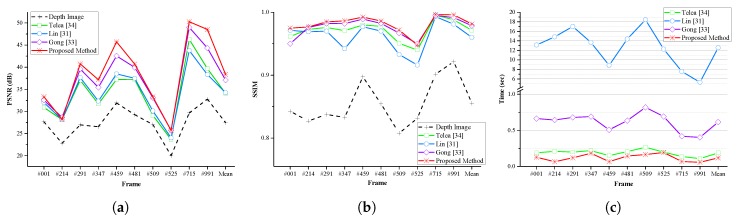
Comparison of (**a**) PSNR; (**b**) SSIM; and (**c**) running time on the selected Tsukuba Stereo Dataset.

To evaluate the GrowFill values given by the proposed method, we used the Tsukuba Stereo Dataset. This dataset provides a total of 1800 image pairs including color, ground truth depth (disparity), and occlusion images. The experiments were conducted using both the color images and occluded depth images. The occluded depth images are generated by excluding the occlusion regions from the ground truth depth. In the dataset, all image pairs are based on the right camera, and the color images are illuminated in daylight. We compared our method with the techniques developed by Telea [34], Lin [31], and Gong [33]. The results of Lin’s method [31] are reported in the corresponding paper. Unless specified otherwise, the neighborhood system of our method was implemented with Moore’s system. The numerical results are evaluated in terms of the peak signal-to-noise ratio (PSNR) [60] in decibels (dB), the structural similarity (SSIM) [61] against the ground truth, and the runtime in seconds (s). The runtime is averaged over 10 repeated experiments of our implementation in the C language. Ten different image pairs (frame numbers 1, 214, 291, 347, 459, 481, 509, 525, 715 and 991) were selected [31] and both quantitative and qualitative tests were performed. Figure 12 presents the visual results of the qualitative evaluation, and Table 2 and Figure 13 illustrate the results of the quantitative evaluation. The results obtained from each method show that the proposed method gives better performance than the previous techniques on both the quantitative and qualitative evaluations. The proposed method gives the best performance in all but two cases in the quantitative evaluation results. Frame number 214 (PSNR of Gong’s method [33] is 0.425 dB higher than that of the proposed method) and frame number 525 (SSIM of Gong’s method [33] is about 0.002 higher than that of the proposed method). In particular, the proposed method is the fastest among those compared here for all selected datasets. On average, for the selected dataset, the proposed method improves the PSNR by 10.898 dB, whereas the methods of Telea [34], Lin [31], and Gong [33] produce improvements of 6.627 dB, 6.772 dB, and 9.620 dB, respectively. Our method improves the SSIM value by 0.126, compared with enhancements of 0.116, 0.105, and 0.124, respectively, for the other approaches. The average runtime of the proposed method is 0.118 s, faster than that of Telea’s method [34] (0.187 s) and Gong’s method [33] (0.615 s), and considerably quicker than Lin’s method [31] (12.543 s).

Table 3 presents the experimental results using the entire Tsukuba Stereo Dataset. In this experiment, the proposed method was compared with the methods of Telea [34] and Gong [33], which represent the fastest and best performing methods among those compared in the previous experiments, respectively. Additionally, we implemented the proposed method with both the Moore and von Neumann neighborhood systems. It is clear that the proposed method outperforms the compared methods. On average, for the entire dataset, the proposed method with the Moore and von Neumann neighborhood systems improves the PSNR by 14.485 dB and 14.067 dB and enhances the SSIM value by 0.116 and 0.115 in 0.138 s and 0.057 s, respectively. The methods of Telea [34] and Gong [33] improve the PSNR by 10.691 dB and 13.298 dB and the SSIM value by 0.109 and 0.114 in 0.117 s and 0.544 s, respectively. In particular, the proposed method with Moore’s neighborhood system achieves the best results in terms of PSNR and SSIM, and the proposed method with the von Neumann neighborhood system is the fastest. From these results, we observe that the proposed method performs best among all compared methods, regardless of the neighborhood system used.

**Figure 14:**
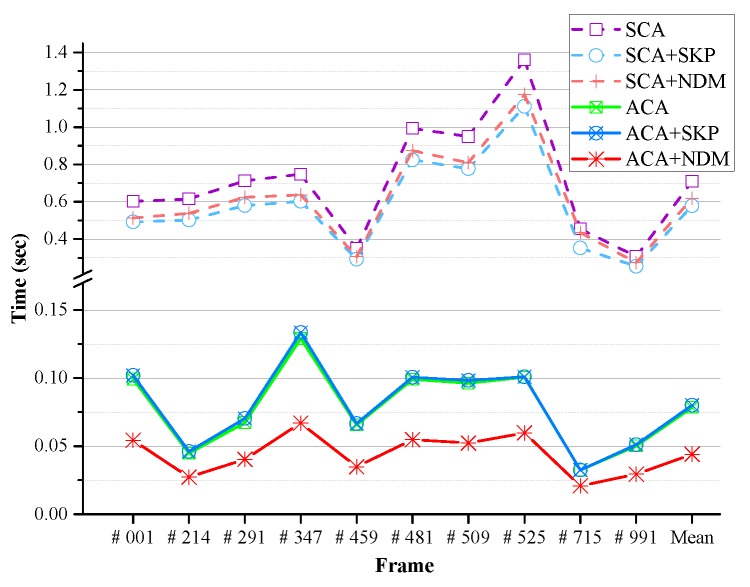
Comparison of runtimes using the selected Tsukuba Stereo Dataset.

In addition, we compared the performance of the internal algorithms of the proposed method (GrowFill) to verify the effects of the ACA and the NDM. Table 4 and Table 5 present the quantitative results for both SCA- and ACA-based methods with Moore’s neighborhood system on the selected Tsukuba Stereo Dataset, respectively. In the experiments, the NDM of our method was compared with the skipping method (SKP) suggested in [62] to reduce the computational cost. We can see that the PSNR, SSIM, and number of iterations of the algorithms did not deteriorate with the SKP or NDM schemes. However, the runtime is reduced by using the schemes. The pure ACA-based method is about 4.4 times faster than the pure SCA-based method. Nonetheless, the proposed method based on ACA combined with NDM is about 1.3-times faster than the pure ACA-based method, and there is no fall-off in quality. As a result, the proposed method (ACA + NDM) is about six-times faster than the pure SCA-based method. The method based on ACA combined with SKP is slower than the pure ACA-based method, although the method based on SCA combined with SKP is faster than the pure SCA-based method. From these results, we can observe that SKP works faster based on SCA, not on the ACA. In the ACA-based experiments, the method with NDM is about 1.4-times faster than the ACA-based method with SKP. Figure 14 compares the runtimes of each internal algorithm. In all cases, the ACA-based methods are faster than the SCA-based methods. Further, the proposed method (ACA + NDM) is the fastest. The results in the tables show that the pure ACA-based method requires only one-third of the number of iterations in the SCA-based method under the same experimental conditions. Note that the runtime can only be reduced by reducing the number of iterations. In the Appendix A, the results obtained with the von Neumann neighborhood system are described in detail.

Table 6 compares the internal algorithms of our method with the Moore and von Neumann neighborhood systems on the entire Tsukuba Stereo Dataset. We can see that the proposed method (ACA + NDM) with the Moore and von Neumann neighborhood system is about 6.5 and 8 times faster than the pure SCA-based method, though the PSNR decreases slightly (by about 0.09 and 0.105 dB, respectively).

The results of the comparison between the RGB and Lab color spaces are presented in Table 7. The experiments show that the PSNR and SSIM performance is improved, and the number of iterations and runtime are decreased, by transforming from the RGB to Lab color space. Thus, the change of color space is an effective means of improving the performance of the algorithm.

**Figure 15:**
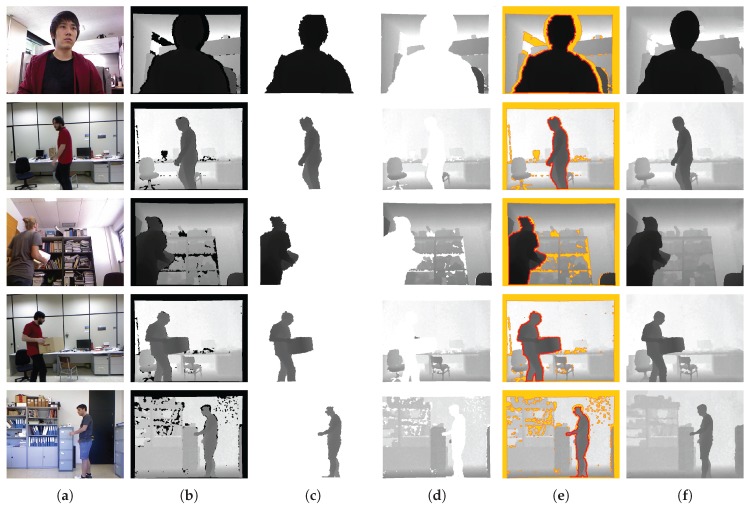
Examples of depth enhancement using the proposed method. (**a**,**b**) are the color and original depth images, respectively; (**c**,**d**) are the object and background depth regions obtained by the proposed method, respectively; (**f**) shows the enhanced depth images obtained by the GrowFill algorithm using (**e**) as the input depth image; the yellow marker in (**e**) indicates the original depth holes; red and orange markers in (**e**) show the expanded regions by using the morphological operations based on (**c**,**d**), respectively.

**Figure 16:**
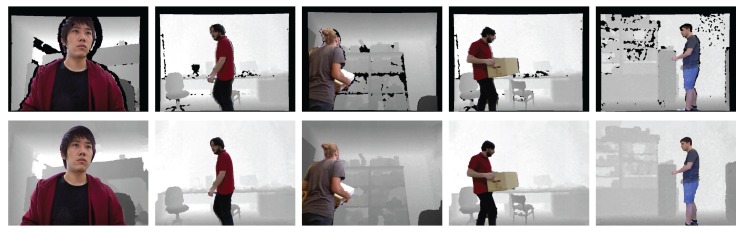
The synthesized object image by using the object depth regions. Top row is based on the original depth image (Figure 15b). Bottom row is based on the enhanced depth images (Figure 15f).

**Figure 17:**
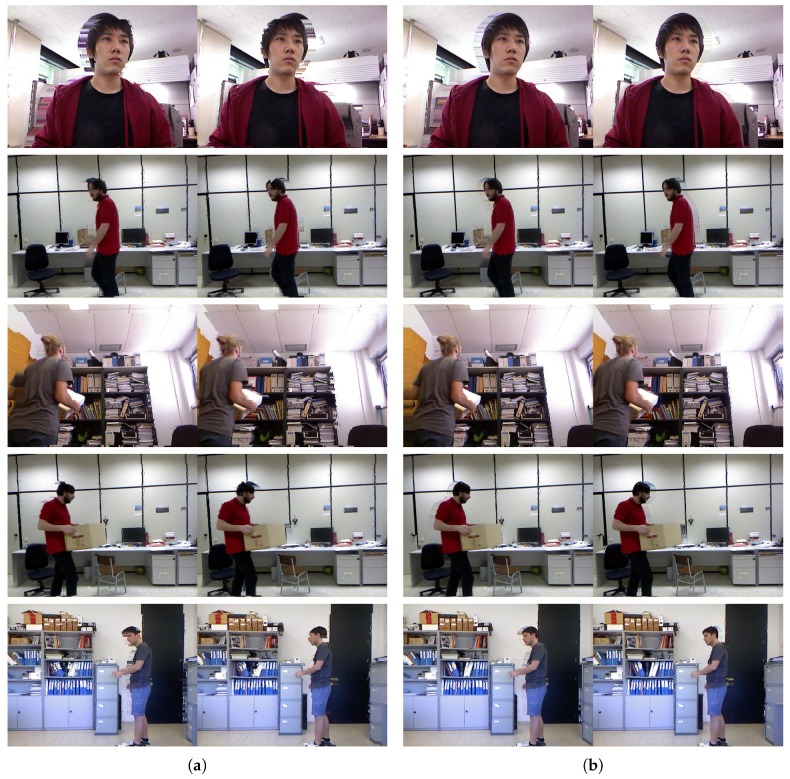
Comparison of the quality of the stereoscopic images. (**a**,**b**) are generated using original and enhanced depth images by the proposed method, respectively.

Finally, we conducted experiments on the real-world dataset [43,55] and our own dataset to verify the effectiveness of our enhancement method. For the depth normalization, we set $Z_{A}=0.4$ m and $Z_{B}=3$ m (near range) for our data and $Z_{A}=0.8$ m and $Z_{B}=4$ m (default range) for the dataset in [43,55]. The extracted object (Figure 15c) and background (Figure 15d) regions were utilized to recover accurate depth information around the object. By taking advantage of the extracted object regions and morphological operations, depth regions around the object were set as the estimable regions in the GrowFill. The yellow marker in Figure 15e indicates the original depth holes. The red and orange markers in Figure 15e indicate the expanded depth holes by using the morphological operations on the object and background regions, respectively. The disk-shaped kernels with r=6 for the object and r=3 for the background regions were used in the morphology. The reason for expanding the depth hole is to recover the correct depth information by removing the incorrect depth information in the original depth image as shown in Figure 16, top row, in which the color regions indicate the corresponding object depth regions and it can be noticed that the background also appears in the object depth regions. Figure 15f shows the enhanced depth image processed by the proposed method using Figure 15e as the input image, from which we can easily observe that the quality of the depth image has improved compared with the original depth images (Figure 15b). In particular, not only are the depth values of the depth images complete but the object boundaries have also been clearly recovered. The enhanced depth images (Figure 16, bottom row) shows that the object shape is more accurate than the original depth images (Figure 16, top row). In addition, the results in Figure 17 were obtained by applying the DIBR technique to generate stereoscopic images with background pixel extrapolation on newly exposed regions after 3D image warping. Figure 17b shows the visual enhancement given by the proposed method.

## 4. Conclusions

The main goal of this study was to enhance the quality of depth efficiently. To achieve this goal, a new depth enhancement approach has been introduced. The proposed method consists of an image segmentation algorithm to extract object regions and a weighted linear combination of spatial filtering algorithms. For inner holes, the characteristics of the hole regions inside the object regions were considered, and for other hole regions, an ACA-based depth recovery algorithm was combined with NDMs. Compared with the initial depth image, our experimental results on the Tsukuba Stereo Dataset show an improvement of 14.485 dB in PSNR and 0.116 in SSIM with Moore’s neighborhood system with an average runtime of only 0.138 s. With the von Neumann neighborhood system, our method achieves improvements of 14.067 dB in PSNR and 0.115 in SSIM in 0.057 s. Comparative experiments show that our method outperforms all compared approaches in terms of both quantitative and qualitative evaluations. Moreover, through experiments with a real-world dataset, we have confirmed that the object shape is recovered and the performance is improved. It is important to note that the proposed method is efficient enough to be employed in near-real-time applications, and it is expected that object regions extracted using our image segmentation algorithm could easily be utilized for activities such as view synthesis and virtual conference systems.

## Figures and Tables

**Table 1:** Quantitative evaluation results using Fernandez’s Kinect dataset. Red text indicates the best, and green text indicates the second best $F_{1}$ score. MOG, mixture of Gaussians; CB, codebook; DECB, depth-extended codebook.

	Wall	Hallway	Chair Box		Shelves	Global	
Method	# 93	# 120	# 278	# 286	# 197	Mean	Std
**MOG4D**	0.406	0.424	0.883	0.865	0.927	0.701	0.262
**CB1D**	0.927	0.791	0.904	0.904	0.897	0.885	0.054
**CB4D**	0.843	0.606	0.936	0.907	0.855	0.829	0.131
**DECB**	0.966	0.782	0.937	0.928	0.926	0.908	0.072
**Ours**	0.930	0.800	0.907	0.911	0.950	0.900	0.058

**Table 2:** Quantitative evaluation results on the selected Tsukuba Stereo Dataset. Red text indicates the best, and green text indicates the second best performance. PSNR, peak signal-to-noise ratio; SSIM, structural similarity.

Frame	Depth Image		Telea [34]			Lin [31]			Gong [33]			Proposed Method		
	PSNR	SSIM	PSNR	SSIM	Time	PSNR	SSIM	Time	PSNR	SSIM	Time	PSNR	SSIM	Time
**# 001**	27.659	0.843	30.912	0.962	0.188	32.036	0.950	13.125	32.579	0.971	0.663	33.314	0.975	0.127
**# 214**	22.824	0.827	28.138	0.973	0.209	28.141	0.969	14.833	28.657	0.976	0.643	28.232	0.977	0.065
**# 291**	26.972	0.838	37.056	0.976	0.198	37.665	0.970	17.013	39.590	0.982	0.679	40.730	0.985	0.118
**# 347**	26.549	0.833	31.699	0.971	0.218	32.343	0.942	13.666	35.435	0.982	0.691	37.177	0.986	0.181
**# 459**	31.920	0.897	37.222	0.980	0.149	38.480	0.977	8.858	42.535	0.989	0.507	45.723	0.992	0.066
**# 481**	29.272	0.854	37.192	0.978	0.206	37.488	0.970	14.389	39.944	0.982	0.634	40.691	0.986	0.143
**# 509**	27.038	0.808	29.132	0.951	0.264	30.120	0.933	18.415	33.096	0.967	0.818	33.299	0.972	0.163
**# 525**	20.006	0.832	23.665	0.940	0.198	24.044	0.916	12.270	25.731	0.950	0.692	25.732	0.948	0.191
**# 715**	29.665	0.902	46.269	0.995	0.136	43.772	0.993	7.555	48.984	0.995	0.417	50.255	0.996	0.067
**# 991**	32.781	0.921	39.666	0.988	0.107	38.312	0.981	5.309	44.331	0.992	0.402	48.511	0.996	0.056
**Mean**	27.468	0.855	34.095	0.971	0.187	34.240	0.960	12.543	37.088	0.979	0.615	38.366	0.981	0.118

**Table 3:** Quantitative evaluation results using the Tsukuba Stereo Dataset. The best performance is highlighted in bold.

Method	Mean		
	PSNR	SSIM	Time
**Depth Image**	26.762	0.871	-
**Telea [34]**	37.453	0.980	0.117
**Gong [33]**	40.060	0.985	0.544
**Ours (von Neumann)**	40.829	0.986	**0.057**
**Ours (Moore)**	**41.247**	**0.987**	0.138

**Table 4:** Quantitative evaluation results for comparing internal algorithms of the ACA-based method on the selected Tsukuba Stereo Dataset. The best performance is highlighted in bold. SCA, synchronous cellular automata; SKP, skipping method; NDM, neighborhood distance map.

Frame	PSNR			SSIM			Iterations			Time		
	SCA	SCA	SCA	SCA	SCA	SCA	SCA	SCA	SCA	SCA	SCA	SCA
		+ SKP	+ NDM		+ SKP	+ NDM		+ SKP	+ NDM		+ SKP	+ NDM
**# 001**	33.313	33.313	33.313	0.975	0.975	0.975	118	118	118	0.602	**0.493**	0.513
**# 214**	28.232	28.232	28.232	0.977	0.977	0.977	131	131	131	0.615	**0.503**	0.538
**# 291**	40.730	40.730	40.730	0.985	0.985	0.985	142	142	142	0.712	**0.579**	0.624
**# 347**	37.173	37.173	37.173	0.986	0.986	0.986	147	147	147	0.748	**0.603**	0.637
**# 459**	45.783	45.783	45.783	0.993	0.993	0.993	89	89	89	0.351	**0.292**	0.305
**# 481**	40.693	40.693	40.693	0.986	0.986	0.986	211	211	211	0.993	**0.824**	0.874
**# 509**	33.299	33.299	33.299	0.972	0.972	0.972	164	164	164	0.949	**0.778**	0.810
**# 525**	25.732	25.732	25.732	0.948	0.948	0.948	254	254	254	1.361	**1.111**	1.174
**# 715**	49.491	49.491	49.491	0.996	0.996	0.996	130	130	130	0.454	**0.353**	0.434
**# 991**	48.516	48.516	48.516	0.996	0.996	0.996	88	88	88	0.308	**0.255**	0.274
**Mean**	38.296	38.296	38.296	0.981	0.981	0.981	147.4	147.4	147.4	0.709	**0.579**	0.619

**Table 5:** Quantitative evaluation results for comparing internal algorithms of the proposed method on the selected Tsukuba Stereo Dataset. The best performance is highlighted in bold. ACA, asynchronous cellular automata.

Frame	PSNR			SSIM			Iterations			Time		
	ACA	ACA	ACA	ACA	SCA	ACA	ACA	ACA	ACA	ACA	ACA	ACA
		+ SKP	+ NDM		+ SKP	+ NDM		+ SKP	+ NDM		+ SKP	+ NDM
**# 001**	33.314	33.314	33.314	0.975	0.975	0.975	50	50	50	0.178	0.181	**0.127**
**# 214**	28.232	28.232	28.232	0.977	0.977	0.977	26	26	26	0.088	0.090	**0.065**
**# 291**	40.730	40.730	40.730	0.985	0.985	0.985	44	44	44	0.158	0.160	**0.118**
**# 347**	37.177	37.177	37.177	0.986	0.986	0.986	68	68	68	0.242	0.248	**0.181**
**# 459**	45.723	45.723	45.723	0.992	0.992	0.992	37	37	37	0.097	0.098	**0.066**
**# 481**	40.691	40.691	40.691	0.986	0.986	0.986	60	60	60	0.198	0.203	**0.143**
**# 509**	33.299	33.299	33.299	0.972	0.972	0.972	51	51	51	0.219	0.224	**0.163**
**# 525**	25.732	25.732	25.732	0.948	0.948	0.948	66	66	66	0.255	0.262	**0.191**
**# 715**	50.255	50.255	50.255	0.996	0.996	0.996	38	38	38	0.084	0.084	**0.067**
**# 991**	48.511	48.511	48.511	0.996	0.996	0.996	39	39	39	0.085	0.085	**0.056**
**Mean**	38.366	38.366	38.366	0.981	0.981	0.981	47.9	47.9	47.9	0.160	0.164	**0.118**

**Table 6:** Quantitative evaluation results for comparing internal algorithms of the proposed method on the entire Tsukuba Stereo Dataset. Left and right tables show the results using the Moore and von Neumann systems, respectively. The best computation times are highlighted in bold.

Moore	Mean				von Neumann	Mean			
Method	PSNR	SSIM	Iterations	Time	Method	PSNR	SSIM	Iterations	Time
**Depth Image**	26.762	0.871	-	-	**Depth Image**	26.762	0.871	-	-
**SCA**	41.337	0.987	197.7	0.902	**SCA**	40.934	0.986	153.1	0.467
**ACA**	41.247	0.987	59.4	0.186	**ACA**	40.829	0.986	51.0	0.095
**SCA + SKP**	41.337	0.987	197.7	0.714	**SCA + SKP**	40.934	0.986	153.1	0.401
**ACA + SKP**	41.247	0.987	59.4	0.189	**ACA + SKP**	40.829	0.986	51.0	0.097
**SCA + NDM**	41.337	0.987	197.7	0.798	**SCA + NDM**	40.934	0.986	153.1	0.412
**ACA + NDM**	41.247	0.987	59.4	0.138	**ACA + NDM**	40.829	0.986	51.0	0.057

**Table 7:** Comparison of quantitative evaluation results for color space on the entire Tsukuba Stereo Dataset. The best performance is highlighted in bold.

Method	Mean			
	PSNR	SSIM	Iterations	Time
**Depth Image**	26.762	0.871	-	-
**SCA (RGB)**	41.290	0.986	211.6	0.978
**SCA (Lab)**	**41.337**	**0.987**	**197.7**	**0.902**
**ACA + NDM (RGB)**	41.198	0.986	63.4	0.150
**ACA + NDM (Lab)**	**41.247**	**0.987**	**59.4**	**0.138**

## Appendix A

We compared the performance of the internal algorithms of the proposed method with the von Neumann neighborhood system to verify the effects of the ACA and the NDM. The quantitative performance of SCA and ACA-based methods with the von Neumann system on the selected Tsukuba Stereo Dataset is presented in Table A1 and Table A2, respectively. The pure ACA-based method is about 4.3-times faster than the pure SCA-based method. Nonetheless, the proposed method based on ACA combined with NDM is about 1.8-times faster than the pure ACA-based method without any degradation in quality. As a result, the proposed method (ACA + NDM) is about 7.7-times faster than the pure SCA-based method.

**Table A1:** Quantitative evaluation results for comparing internal algorithms of the ACA-based method with von Neumann neighborhood system on the selected Tsukuba Stereo Dataset. The best performance is highlighted in bold.

Frame	PSNR			SSIM			Iterations			Time		
	SCA	SCA	SCA	SCA	SCA	SCA	SCA	SCA	SCA	SCA	SCA	SCA
		+ SKP	+ NDM		+ SKP	+ NDM		+ SKP	+ NDM		+ SKP	+ NDM
**# 001**	32.987	32.987	32.987	0.973	0.973	0.973	79	79	79	0.272	0.239	**0.222**
**# 214**	28.210	28.210	28.210	0.976	0.976	0.976	96	96	96	0.312	0.275	**0.264**
**# 291**	39.993	39.993	39.993	0.983	0.983	0.983	101	101	101	0.339	0.296	**0.289**
**# 347**	36.986	36.986	36.986	0.986	0.986	0.986	120	120	120	0.413	0.360	**0.335**
**# 459**	42.733	42.733	42.733	0.991	0.991	0.991	64	64	64	0.183	0.162	**0.159**
**# 481**	40.221	40.221	40.221	0.985	0.985	0.985	153	153	153	0.490	0.432	**0.407**
**# 509**	33.009	33.009	33.009	0.970	0.970	0.970	104	104	104	0.396	0.344	**0.322**
**# 525**	25.073	25.073	25.073	0.946	0.946	0.946	162	162	162	0.569	0.490	**0.471**
**# 715**	49.741	49.741	49.741	0.997	0.997	0.997	94	94	94	0.244	**0.208**	0.225
**# 991**	48.372	48.372	48.372	0.996	0.996	0.996	63	63	63	0.165	**0.147**	0.149
**Mean**	37.733	37.733	37.733	0.980	0.980	0.980	103.6	103.6	103.6	0.338	0.295	**0.284**

**Table A2:** Quantitative evaluation results for comparing internal algorithms of the proposed method with von Neumann neighborhood system on the selected Tsukuba Stereo Dataset. The best performance is highlighted in bold.

Frame	PSNR			SSIM			Iterations			Time		
	ACA	ACA	ACA	ACA	SCA	ACA	ACA	ACA	ACA	ACA	ACA	ACA
		+ SKP	+ NDM		+ SKP	+ NDM		+ SKP	+ NDM		+ SKP	+ NDM
**# 001**	32.987	32.987	32.987	0.973	0.973	0.973	48	48	48	0.099	0.102	**0.054**
**# 214**	28.210	28.210	28.210	0.976	0.976	0.976	21	21	21	0.045	0.046	**0.027**
**# 291**	39.981	39.981	39.981	0.983	0.983	0.983	31	31	31	0.067	0.070	**0.040**
**# 347**	36.986	36.986	36.986	0.986	0.986	0.986	62	62	62	0.129	0.133	**0.067**
**# 459**	42.706	42.706	42.706	0.990	0.990	0.990	42	42	42	0.066	0.067	**0.035**
**# 481**	40.221	40.221	40.221	0.985	0.985	0.985	51	51	51	0.099	0.101	**0.055**
**# 509**	33.009	33.009	33.009	0.970	0.970	0.970	38	38	38	0.096	0.098	**0.052**
**# 525**	25.073	25.073	25.073	0.946	0.946	0.946	44	44	44	0.101	0.101	**0.060**
**# 715**	49.412	49.412	49.412	0.997	0.997	0.997	21	21	21	0.033	0.032	**0.021**
**# 991**	48.370	48.370	48.370	0.996	0.996	0.996	37	37	37	0.050	0.051	**0.029**
**Mean**	37.696	37.696	37.696	0.980	0.980	0.980	39.5	39.5	39.5	0.078	0.080	**0.044**
